# Correlations between apparent diffusion coefficient values of WB-DWI and clinical parameters in multiple myeloma

**DOI:** 10.1186/s12880-021-00631-2

**Published:** 2021-06-08

**Authors:** Bei Zhang, Bingyang Bian, Zhiwei Zhao, Fang Lin, Zining Zhu, Mingwu Lou

**Affiliations:** 1 Shenzhen Clinical Medical School, Guangzhou University of Chinese Medicine, Shenzhen, China; 2 Department of Radiology, First Hospital of Jilin University, Changchun, China; 3 Department of Hand and Foot Surgery, First Hospital of Jilin University, Changchun, China; 4 Department of Radiology, Longgang Central Hospital of Shenzhen, No. 6082, Longgang Road, Longgang District, Shenzhen, 518116 Guangdong Province China

**Keywords:** Apparent diffusion coefficient, Diffusion-weighted imaging, FISH, Multiple myeloma, Therapeutic response

## Abstract

**Background:**

Whole-body diffusion-weighted imaging (WB-DWI) is a method for evaluating bone marrow infiltration in multiple myeloma (MM). This study seeks to elucidate the correlation between the apparent diffusion coefficient (ADC) value and some selected clinical parameters.

**Methods:**

A total of 101 Chinese patients with MM who had undergone WB-DWI from May 2017 to May 2019 were enrolled in this study. The ADC values of the MM lesions and the clinical parameters were quantified at the first (baseline) visit and after four-course induction chemotherapy. Multiple linear regression and logistic analyses were carried out to find the implicit inherent relationships within the patients’ data.

**Results:**

The paired Wilcoxon test showed that the ADC values at the baseline visit (ADC_0_) were significantly lower than the values after four-course induction chemotherapy (ADC_4 c_) (*p *< 0.001), including different therapeutic responses. The Revised International Staging System (RISS) stage, type of MM, and β2-microglobulin (β2-MG) were predictors of clinically significant increases or decreases in the ADC values (*p* < 0.05). Multiple linear regression showed that the ADC_0_ was negatively associated with β2-MG (*p* < 0.001) and immunoglobulin heavy chain gene rearrangement (*p* = 0.012), while the RISS Stage III (*p* = 0.001), type IgG λ (*p* = 0.005), and albumin were negatively associated with ADC_4 c_ (*p* = 0.010). The impacts of the therapeutic response were associated with ADC_0_ and immunoglobulin heavy chain gene rearrangement (*p* < 0.001).

**Conclusion:**

The ADC values of WB-DWI may be associated with clinical parameters of MM including the fluorescence in situ hybridization result, and may be useful in the prognosis of patients with MM.

*Trial Registration*: ChiCTR2000029587

## Background

Multiple myeloma (MM) is an abnormal proliferation of plasma cells characterized by hypercalcemia, renal insufficiency, anemia, and bone disease [1], which can result in various complications and high mortality [2].

Magnetic resonance (MR) imaging is usually performed to evaluate the burden of disease in patients with MM and to make therapeutic decisions [3]. Although conventional MR imaging analysis has been used for many years to assess the disease burden [4], it cannot fully explain the clinical features of MM intramedullary lesions. Whole-body diffusion-weighted imaging (WB-DWI) is a clinically applicable MRI method that adopts the short tau inversion recovery (STIR) sequence to suppress background signals [5]. However, a fundamental limitation of DWI is the lack of relationship between imaging indicators and clinical or even genetic characteristics. However, the apparent diffusion coefficient (ADC) values may be helpful in overcoming these limitations. The ADC value may likely provide more sensitive biomarkers for detecting changes in the microstructure that occur in the bone marrow, which may be a more accurate biomarker for the prediction of disease prognosis. Although WB-DWI has been applied to MM in some tentative studies [6, 7], its potential application in the estimation of genetic variation in MM is not yet well established.

Abnormal karyotypes are present in 30–50% of MM cases [8]. These include various cytogenetic abnormalities that influence MM risk stratification and prognosis and have become the target of new therapeutic methods. DNA probes using the interphase fluorescence in situ hybridization (iFISH) technology combined with chromosomal analysis overcomes the limitations of the conventional detection of mitotic phase chromosomes and has greatly improved the sensitivity of cytogenetic analysis [9]. High-frequency chromosomal abnormalities detected by iFISH in MM include 1q21, rearrangements at the immunoglobulin heavy chain (IGH) locus, and p53. Some studies have found that these genetic mutations are significantly associated with albumin and globulin levels and affect the prognosis and treatment response of MM [9–11]. Mutations with high levels of albumin and globulin means that the proliferation and secretion of tumor cells are more vigorous, which indirectly means more tumor cells and smaller intercellular spaces. These changes may lead to the limited diffusion of water molecules, relatively high DWI signal and low ADC value. However, the relationship between the ADC values of DWI imaging, which quantitatively reflect the disease burden of MM bone disease, and genetic variation has barely been clarified. Specifically, it needs further verification whether the ADC value of MM bone lesions is related to genetic variation and whether the ADC value can map the microscopic characterization of genetics from the macroscopic point of view.

While cytogenetic risk is helpful in evaluating MM status, it only partially accounts for the variation in outcomes among patients. Current studies suggest that albumin, LDH, and β2-MG may contribute to the risk stratification of MM patients [12, 13]. There is a linear relationship between these indicators and overall survival or progression-free survival. The evidence suggests that a decreased albumin level is a marker of disease burden and that increases in LDH and β2-MG are also markers of invasive disease [14]. It is possible that the baseline RISS stage, LDH, albumin, along with β2-MG and M protein type, are predictive of treatment outcome and could potentially identify patients with a more unfavorable prognosis.

The purpose of this study was to investigate the associations between the ADC values of the bone marrow lesions in patients with MM and FISH results along with other clinical parameters. Our hypothesis was that ADC values have a degree of correlation with genetic variations (FISH results) as well as clinical and laboratory parameters and that this is related to the therapeutic response of MM.

## Methods

### Participants

The study was approved by the Institutional Ethics Committee of the First Hospital of Jilin University, Changchun, China. Written informed consent was obtained from the patients by email or letter. We retrospectively reviewed the Picture Archiving and Communication System for MM patients who had undergone WB-DWI during a 24-month period (May 2017 to November 2019). The inclusion criteria were as follows: (1) patients aged between 18 and 70 years; (2) patients with a diagnosis of MM according to established diagnostic criteria; (3) patients who had undergone WB-DWI performed according to our standard MM protocol; (4) patients who had received autologous stem cell transplantation after induction chemotherapy; (5) patients who had received WB-DWI before and after four courses of induction chemotherapy. There is an interval between induction and transplantation. The time point of the imaging examination after treatment falls within this interval.

The exclusion criteria were as follows: (1) patients with bone diseases other than MM; (2) imaging data with poor quality due to body movements or other artifacts; and (3) the presence of an unexplained high signal outside the skeleton in DWI images. This unexplained high signal mainly refers to an abnormally high DWI signal, the nature of which cannot be determined, even in combination with T1WI and T2 STIR sequences. In addition to the conventional sagittal scans of the spine and local DWI axial scans, local sagittal or axial T1WI and T2 STIR scans were performed on these suspicious lesions to determine whether they were located in the skeleton, especially where the lesion appeared to be in close proximity. While these patients needed to be excluded from this study, additional examinations are required to diagnose the presence of other diseases in addition to MM. The results of these examinations would indicate specific personalized treatment and management programs.

### FISH procedure

Fish results were obtained before induction chemotherapy. Slides were pretreated according to the manufacture's protocol (Vysis, USA). The samples were cultured at 37 ° for about 25 min in low osmotic potassium chloride. After centrifugation, the supernatant was discarded and the residue was kept for use. The FISH probes used in this study included p32, p53, q21, IGH, and RB1 (Vysis). FISH analysis of probe hybridization was performed with a 100 × objective fluorescence microscope (Olympus BX5, Japan) with single and triple emission filters. The iFISH results were described according to the standards of the International System for Human Cytogenetic Nomenclature (ISCN) 2016 [9].

### Relevant clinical parameters

In 2015, based on the International Staging System (ISS), the International Myeloma Working Group (IMWG) developed the Revised International Staging System (RISS) for the inclusion of genetic risk factors and LDH levels. RISS staging was determined as previously described [15]. The type of M proteins present at the time of diagnosis should be recorded. Albumin, LDH, andβ2-MG levels in serum need to be recorded before and after induction chemotherapy. These were measured using a Beckman Coulter AU Analyzer (Beckman Coulter, USA). The time interval between obtaining these records and the WB-DWI examination was less than 3 days. The time interval before and after induction chemotherapy was the same.

### Treatment

All patients received stem cell transplantation after induction chemotherapy. Each patient received chemotherapy as one of the following three options (VCD = bortezomib, cyclophosphamide and dexamethasone; VD = bortezomib and dexamethasone; VAD = vincristine, doxorubicin, and dexamethasone) followed by mobilization chemotherapy with CAD (cyclophosphamide, doxorubicin, and dexamethasone). Treatment response was assessed by the National Comprehensive Cancer Network guidelines for MM, including complete response (CR), strict complete response (sCR), very good partial response (VGPR), partial response (PR), stable disease (SD) and progressive disease (PD) [16].

### MR imaging

WB-DWIs were performed on a 3.0 T Ingenia MR imaging scanner (Philips, The Netherlands) with a rolling table platform, integrated coil, two surface coils, head coil, and neck coil. Multiple-station scanning was used in the protocol. Seven scans were conducted from “head to toe”. The study protocol included fat-suppressed T2-weighted short time inversion recovery (STIR) acquired separately in the coronal plane for the head-neck, chest, abdomen, pelvis, femur, tibia, and feet districts, and successively merged to create a single “whole body” image. STIR was conducted with the following parameters: repetition time (TR)/echo time (TE)/inversion time (TI), 8600/60/170 ms; image matrix, 290 × 310; field of view (FOV), 400 mm; number of excitations (NEX), 4; slice thickness/gap, 3/0 mm; number of slices, 24; and acquisition time, 3 min. T2 STIR sagittal spine images were obtained with the following parameters: TR/TE/TI 8600/60/160 ms; image matrix, 290 × 310; FOV, 420 mm; NEX, 4; slice thickness/gap, 5/0 mm; number of slices, 24; and acquisition time, 2.5 min. T1-weighted spin-echo coronal images were obtained with the following parameters: TR/TE, 476/9.3; image matrix, 384 × 234; FOV, 500 mm. T1-weighted spin-echo spine sagittal images were obtained with the following parameters: TR/TE, 380/10 ms; image matrix, 320 × 265; FOV, 420 mm; slice thickness/gap, 3/0 mm; acquisition time, 2.5 min. Echo-planar DWI coronal images were conducted with the following parameters: b value, 0 and 1000 s/mm^2^; TR/TE/TI, 5200/106/180 ms; image matrix, 128 × 128; FOV, 400 mm; NEX, 4; slice thickness/gap, 3/0 mm; number of slices, 24; and acquisition time, 4 min. If necessary DWI axial images were obtained with the following parameters: TR/TE/TI, 7800/71/160 ms; image matrix, 265 × 205; FOV, 500 mm; NEX, 3; slice thickness/gap, 7/0 mm; acquisition time, 3 min. The images obtained with a value of b = 1000 s/mm^2^ were then reformatted into a maximum intensity projection (MIP) and evaluated by inversion of the grayscale. The total scan time was close to 45 min. The above parameters are shown in Table 1.

**Table 1:** Technical magnetic resonance imaging parameters

	T2 STIR coronal	T2 STIR sagittal	T1 coronal	T1 sagittal	DWI coronal	DWI axial
Number of slices	24	24	24	24	24	36
FOV	400	420	500	420	400	500
Thickness (mm)	3	5	3	5	3	7
TR (ms)	8600	8600	476	380	5200	7800
TE (ms)	60	60	9.3	10	106	71
TI (ms)	170	160			180	160
Image matrix	290 × 310	290 × 310	384 × 234	320 × 265	128 × 128	265 × 205
Number of excitations	4	4	4	4	4	3
b values (mm^2^/s)					0/1000	0/1000
Acquisition time (s)	3	2.5	3	2.5	4	3

*FOV* field of view, *STIR* short time inversion recovery, *TE* echo time, *TI* inversion time, *TR* repetition time

### Data processing

**Fig. 1:**
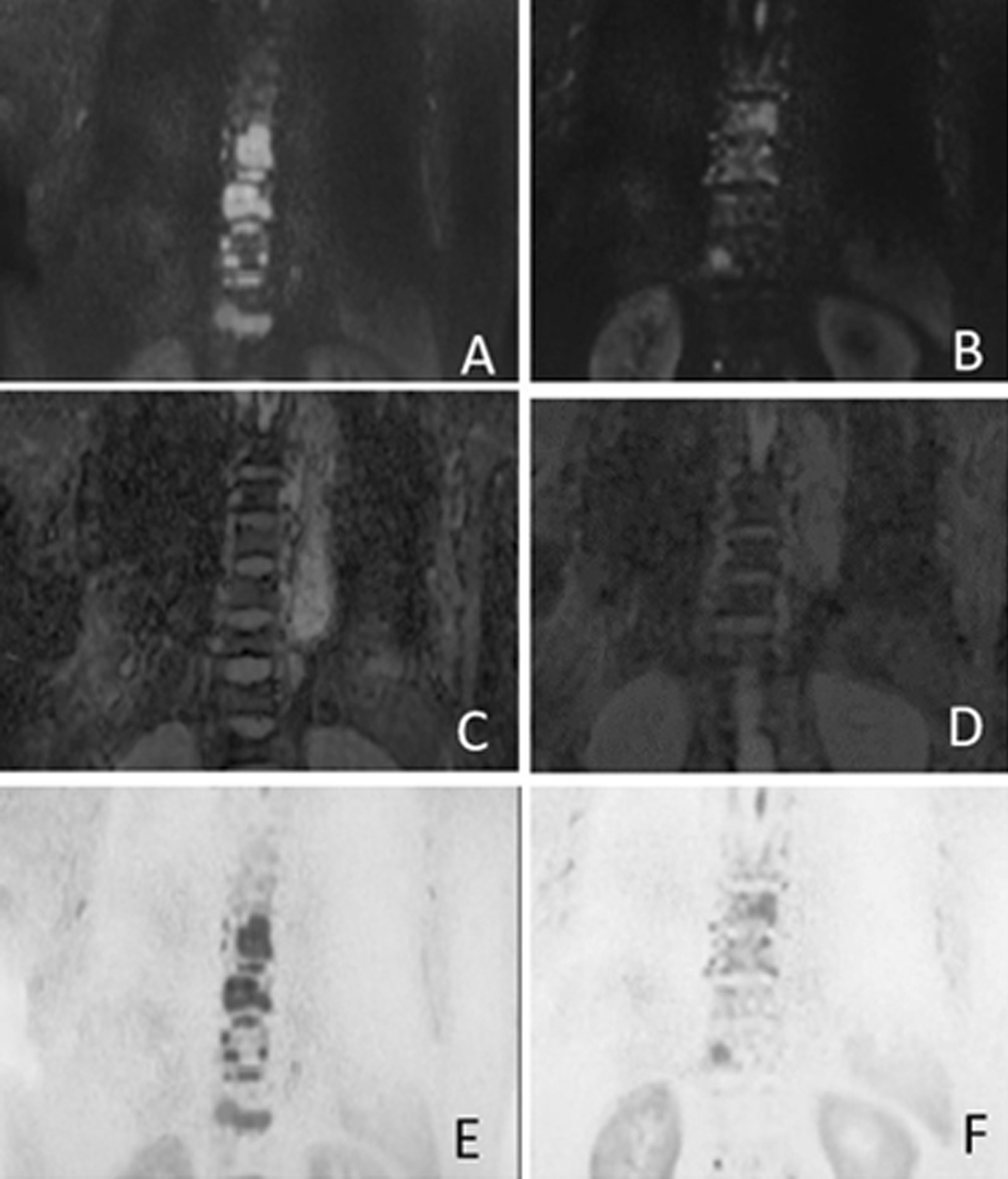
The patient underwent WB-DWI after the diagnosis of multiple myeloma. **A** (DWI b = 1000 s/mm^2^), **C** (ADC map) and **E** (inverted MIP image) show MM lesions of spine at baseline visit. **B** (DWI b = 1000 s/mm^2^), **D** (ADC map) and **F** (inverted MIP image) show MM lesions after 4-course chemotherapy. The ADC value of the lesion in the third lumbar vertebra increased from (0.698 × 10^–3^ mm^2^/s) to (0.796 × 10^–3^ mm^2^/s). In b = 1000 s/mm^2^ DWI images, the lesion area in **B** after treatment was significantly smaller than that in **A** before treatment. This contrast is more intuitive on the inverted image, which is shown in **F** after treatment and **E** before treatment

**Fig. 2:**
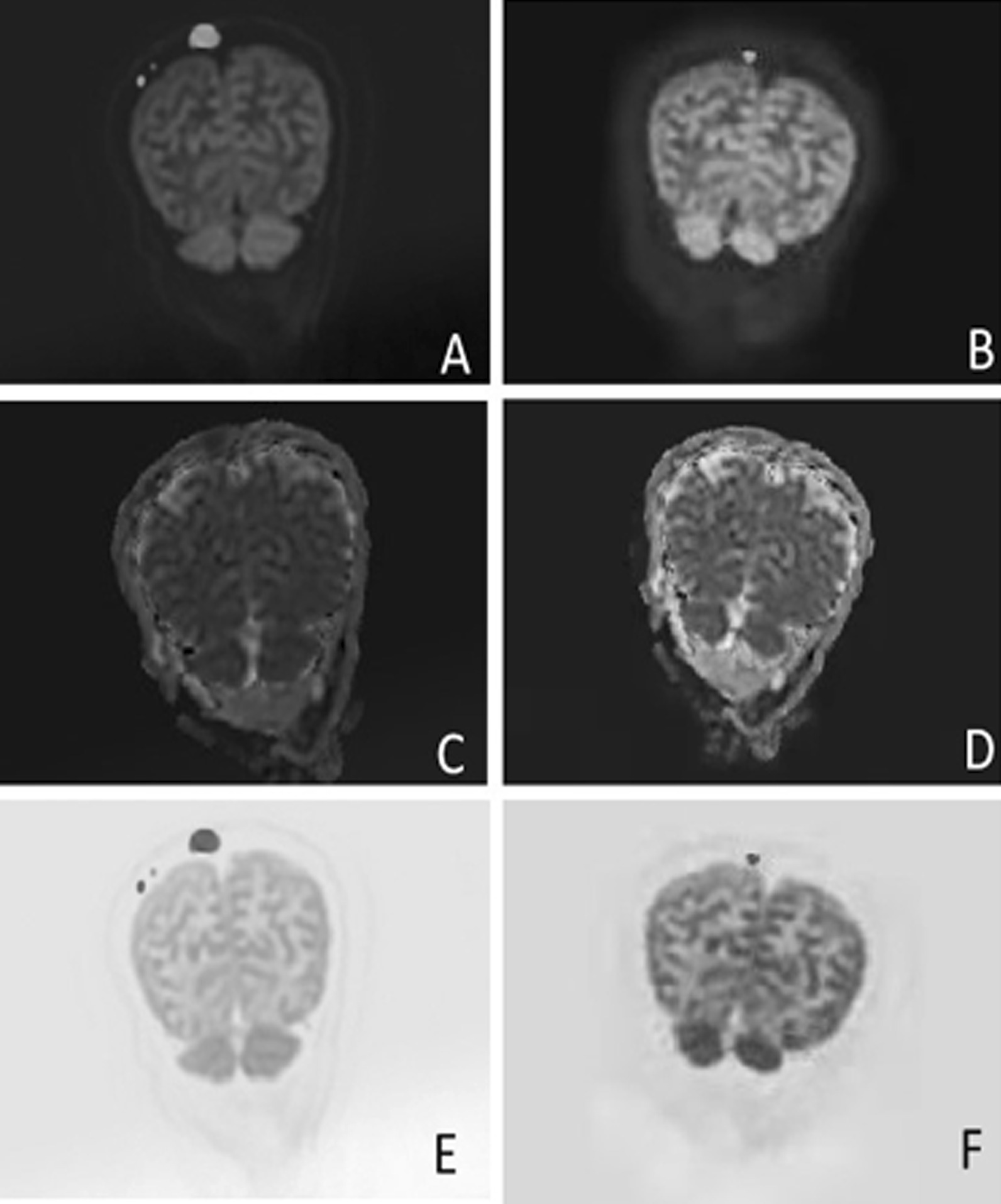
The same patient as Fig. 1, **A** (DWI b = 1000 s/mm^2^), **C** (ADC map) and **E** (inverted MIP image) show MM lesions of skull at baseline visit. **B** (DWI b = 1000 s/mm^2^), **D** (ADC map) and **F** (inverted MIP image) show MM lesions after 4-course chemotherapy. The ADC value of the large lesion in right parietal bone increased from (0.798 × 10^–3^ mm^2^/s) to (0.833 × 10^–3^ mm^2^/s). The change of lesion sizes are more obvious on inverted images (**E** before treatment; **F** after treatment) than that on DWI images (**A** before treatment; **B** after treatment)

The ADC values were measured in six areas (skull, cervical vertebrae, thoracic vertebrae, lumbar vertebrae, bilateral ilium, ribs) on the ADC map. Using region of interest (ROI) analysis, we measured the ADC_0_ and ADC_4_c of the entire cross-sectional area of all the lesions. The ROI is defined as follows: the abnormal signal is recognized in the DWI image combining T1WI and T2 STIR, and the contour is drawn in the corresponding position of the ADC map. The specific outline of the ROI includes all slices of each lesion on the ADC map. Each layer of the lesion should be outlined completely, a ROI should be obtained for each layer. The ADC value is automatically measured for each ROI, and the average value of all these ADC values is regarded as the ADC value of this lesion. The software automatically outlines the lesions, including all the layers showing the lesions on the ADC map (Figs. 1, 2). When the automatic sketch is not accurate, manual modification of the accurate contour of the lesion is required. When the lesion is diffuse, all the levels of the whole bone need to be outlined. The average ADC value of all lesions in each region is the ADC value in that region. If there is no MM lesion in an area, then that area will not be measured, and it will not be included in the calculation of the final ADC value. We generated six values in each subject. For the total of 101 patients, each patient had six sections included in the ADC measurement, resulting in 606 sections altogether. Based on the actual lesion distribution area, only 517 sections were finally included for the 101 patients with the remaining 89 sections being excluded because due to an absence of MM bone lesions. All layers of all the measured sections included MM bone lesions. DWI data were processed and the ADC maps were generated using an EWS workstation (Philips, The Netherlands). The delineation of ROI before and after treatment ensures the consistency of measurement location to the greatest extent. The anatomical position of the lesions before and after treatment was precisely located by using T2-STIR and DWI sequence, and then the ROI of the lesions was sketched by software in the ADC map. The ROI measured by the ADC value of each lesion includes all slices of the lesion. The ROI Delineation and the ADC value was obtained by three attending radiologists, following which the average of the three ADC values was taken to exclude the variability among observers.

The results were analyzed by SPSS software (Version 14.0, IBM, Armonk, New York). The ADC_0_ and ADC_4 c_ including different therapeutic responses were examined with the paired Wilcoxon test. The level of plasma cell infiltration, albumin, LDH, and β2-MG were examined with the paired *t* test. Multiple linear regression and logistic analysis were used to determine the relationships between the ADC values and the clinical variables (age, plasma cell infiltration, RISS Stage and type, albumin, LDH, and serum β2-MG). The impacts of the therapeutic response were investigated using multiple logistic regression. *p* values of < 0.05 were considered statistically significant.

## Results

### Demographic and clinical characteristics

**Fig. 3:**
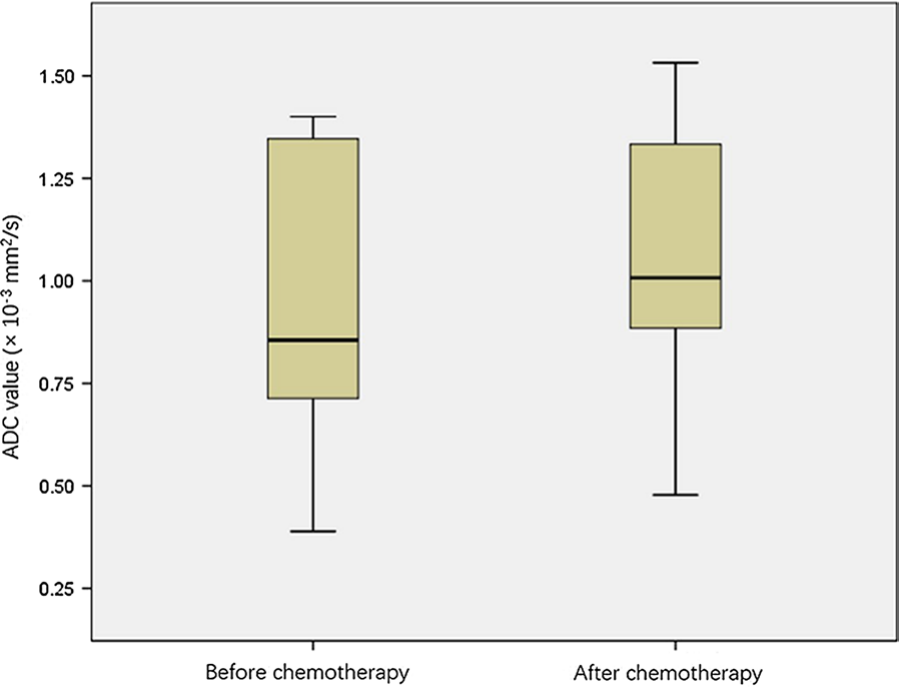
Box plot showed ADC value before and after treatment

**Fig. 4:**
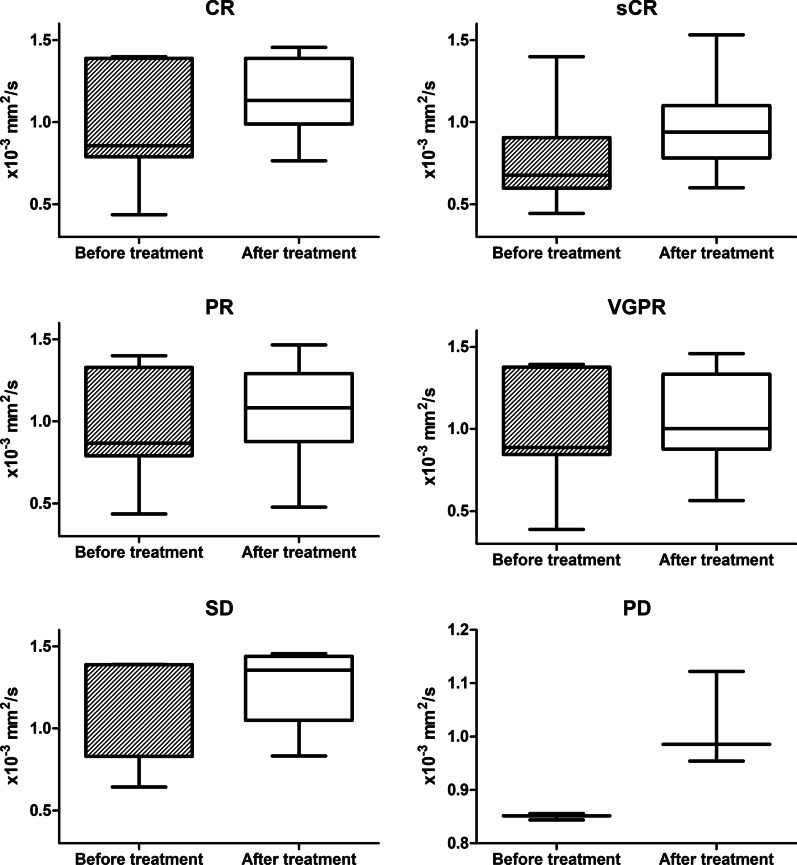
Box plot showed ADC value of before and after treatment different therapeutic responses

**Fig. 5:**
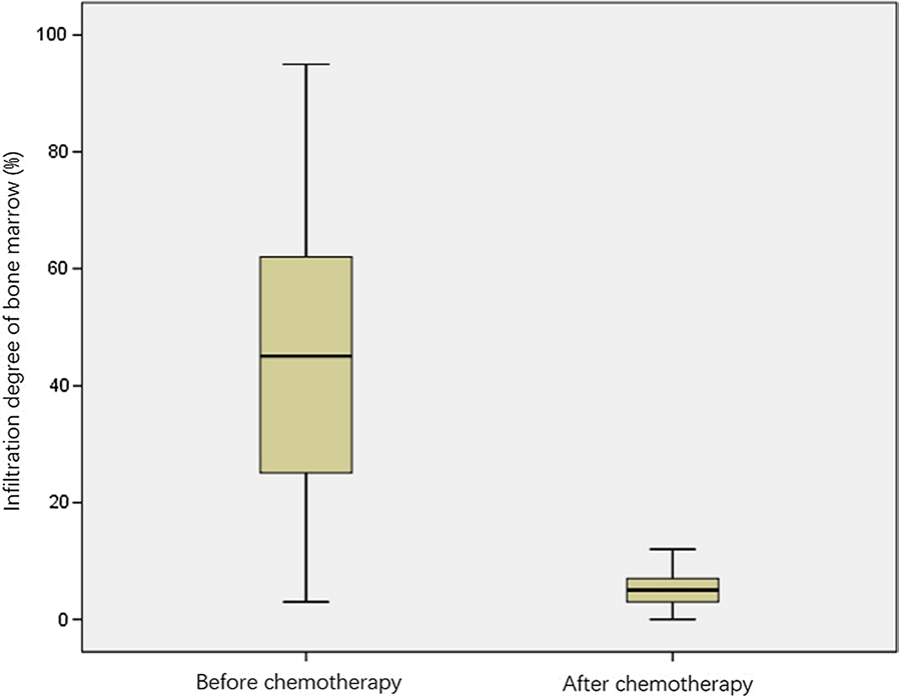
Box plot showed Infiltration degree of bone marrow before and after treatment

A total of 101 patients with MM were included in the study. Table 2 shows their demographic and clinical characteristics including the RISS staging and type of the entire cohort at the baseline visit. MM in this study affected men more often than women. Table 3, Figs. 3 and 4 show the analysis of variance of the mean ADC values in patients including different therapeutic responses before and after chemotherapy. The ADC_0_ was significantly lower than the ADC_4c_ (*p* < 0.001). Plasma cell infiltration at baseline was significantly higher than after chemotherapy (Table 3 and Fig. 5).

**Table 2:** Baseline demographics and clinical characteristics of the patient population

Parameters	Baseline demographics and clinical characteristics
No. of subjects	101
Sex (M/F)	63/38 (62.4%/37.6%)
Age (mean) (year)	57.06 ± 9.72
RISS stage	
Stage I	11 (10.9%)
Stage II	52 (51.5%)
Stage III	38 (37.6%)
Type	
IgA λ	11 (10.9%)
IgD λ	8 (7.9%)
IgG λ	31 (30.7%)
λ	19 (18.8%)
IgA κ	11 (10.9%)
IgG κ	15 (14.9%)
κ	6 (5.9%)
Therapeutic response	
CR	35 (34.7%)
sCR	22 (21.8%)
PR	20 (19.8%)
VGPR	15 (14.9%)
SD	6 (5.9%)
PD	3 (3%)

*CR* complete response, *PD* progressive disease, *PR* partial response, *RISS* revised international staging system, *sCR* strict complete response, *SD* sable disease, *VGPR* vary good partial response

**Table 3:** Within-subjects ADC value change after chemotherapy

Parameters	Baseline visit	After 4-course chemotherapy	*p* value
ADC value (× 10^–3^ mm^2^/s)	0.8556 (0.7130–1.361)	1.0076 (0.8817–1.3336)	< 0.001^a^
Therapeutic response			
CR	0.8561 (0.7890–1.3884)	1.1330 (0.9887–1.3883)	< 0.001^a^
sCR	0.6772 (0.5985–0.9066)	0.9756 ± 0.2724	< 0.001^a^
PR	0.8673 (0.7912–1.3301)	1.0961 ± 0.2724	< 0.001^a^
VGPR	0.9555 ± 0.3326	1.0667 ± 0.2537	0.004^a^
SD	1.3883 (0.8290–1.3891)	1.2588 ± 0.2467	0.151
PD	0.8504 ± 0.0602	1.0206 ± 0.8934	0.089
Plasma cell infiltration (%)	44.35 ± 23.11	5.01 ± 7.62	< 0.001^a^
Albumin (g/L)	34.36 ± 7.31	37.44 ± 7.03	< 0.001^a^
LDH (U/L)	222.41 ± 85.29	227.94 ± 48.14	0.570
β2 microglobulin (μg/mL)	8.75 ± 7.15	4.27 ± 3.00	< 0.001^a^

*ADC* apparent diffusion coefficient, *CR* complete response, *LDH*  lactic dehydrogenase, *PD* progressive disease, *PR* partial response, *sCR* strict complete response, *SD* sable disease, *VGPR* vary good partial response

^a^Significant difference

### Imaging data

Logistic regression demonstrated the influence of clinical parameters on increases and decreases in ADC values after chemotherapy, including age (*p* = 0.289), gender (*p* = 0.634), RISS Stage (*p* < 0.001), type (*p* = 0.005), albumin (*p* = 0.619), LDH (*p* = 0.646), β2-MG (*p* = 0.002), p53 deletion (*p* = 0.912), IGH rearrangement (*p* = 0.007), q21 amplification (*p* = 0.997), RB1 deletion (*p* = 0.280), and p32 deletion (*p* = 0.634) (Table 4). Multiple linear regression showed that β2-MG (β = − 0.038) and IGH rearrangement (β = − 0.107) were correlated with ADC_0_ (Table 5). RISS Stage III (β = − 0.107), IgG λ (β = − 0.151) and albumin (β = − 0.009) were correlated with ADC_4c_ (Table 6).

**Table 4:** Logistic regression results evaluating the predictors of clinically meaningful ADC value increase and decrease after chemotherapy

Parameters	ADC values increase (n = 91)^a^	ADC values decrease (n = 10)^a^	*p* value
Age	1.103 (0.920–1.322)	0.907 (0.756–1.087)	0.289
Gender	1.787 (0.164–19.475)	0.560 (0.051–6.089)	0.634
RISS stage	0.153 (0.008–3.074)	2.536 (1.132–5.684)	< 0.001^b^
Type of MM	0.394 (0.176–0.884)	6.542 (0.325–131.52)	0.005^b^
β2-MG	2.406 (1.094–5.294)	0.416 (0.189–0.914)	0.002^b^
Albumin	0.955 (0.795–1.146)	1.048 (0.872–1.258)	0.619
LDH	1.007 (0.977–1.038)	0.993 (0.964–1.023)	0.646
p53 deletion	1.257 (0.022–71.310)	0.796 (0.014–45.143)	0.912
IGH rearrangement	1.036 (0.978–2.012)	1.074 (1.005–1.995)	0.007^b^
q21 amplification	0 (0 to + ∞)	0 (0 to + ∞)	0.997
RB1 deletion	8.963 (0.167–48.145)	0.112 (0.002–5.980)	0.280
p32 deletion	62.549 (0.120–32.659)	0.560 (0.051–6.098)	0.634

*ADC* apparent diffusion coefficient, *LDH* lactic dehydrogenase, *MM* multiple myeloma, *RISS* revised international staging system

^a^The values are given as the odds ratio, with 95% confidence interval in parentheses

^b^Significant difference

**Table 5:** Multiple linear regression results evaluating the effects of clinically meaningful parameters on ADC_0_

Independent variables	β	SE	β′	t	*p* value
Constant	1.298	0.036		35.788	< 0.001
β2 microglobulin	− 0.038	0.003	− 0.752	− 11.782	< 0.001
IGH rearrangement	− 0.107	0.042	− 0.163	− 2.546	0.012

R^2^ = 0.601, Adjusted R^2^ = 0.593, F = 6.481, *p* = 0.012. ADC_0_ = ADC value at baseline visit

**Table 6:** Multiple linear regression results evaluating the effects of clinically meaningful parameters on the ADC_4c_

Independent variables	β	SE	β′	t	*p* value
Constant	1.536	0.140		10.989	< 0.001
RISS Stage III	− 0.166	0.050	− 0.319	− 3.350	0.001
IgG λ	− 0.151	0.053	− 0.277	− 2.883	0.005
Albumin	− 0.009	0.003	− 0.251	− 2.629	0.010

*RISS* revised international staging system

R^2^ = 0.167, Adjusted R^2^ = 0.141 F = 6.910, *p* = 0.010. ADC_4c_ = ADC value at 4-course induction chemotherapy follow-up

Multiple logistic regression showed that the main predictors of treatment effect in this study included ADC_0_ and (IGH) gene rearrangement (Table 7). ORs (and 95% CIs) of treatment response with stratification of ADC_0_ and IGH gene rearrangement. The reference group was Group sCR since their treatment response was better than that in other groups. Compared to ADC values of 0.9 × 10^–3^ mm^2^/s and above, ADC values of less than 0.8 × 10^–3^ mm^2^/s were less likely to produce a VGPR treatment response. Compared to ADC 0.7 × 10^–3^ mm^2^/s and above, ADC values between 0.3 × 10^–3^ and 0.4 × 10^–3^ mm^2^/s (OR 1.268, 95% CI 0.445–1.116) were more likely to produce a PD treatment response. Patients with p53 (OR 1.828, 95% CI 1.905–3.445) and RB1 (OR 1.492, 95% CI 0.067–1.992) deletions were more likely to have a PD treatment response than other genetic variations.

**Table 7:** Factors that may affect induced chemotherapy response of MM from multivariable logistic regression

Characteristics	Treatment response					
	CR	sCR	PR	VGPR	SD	PD
	Odds Ratio (95% confidence interval)					
ADC value (× 10^–3^ mm^2^/s)						
0.3 < ADC ≤ 0.4	1.666 (3.439–8.069)		2.047 (1.063–1.593)	0.496 (0.631–2.789)*	0.136 (0.280–1.068)*	1.268 (0.445–1.116)*
0.4 < ADC ≤ 0.5	1.917 (3.912–9.396)		0.110 (0.016–0.740)*	0.755 (0.755–1.227)*	1.811 (1.961–6.727)	0.496 (0.208–2.398)
0.5 < ADC ≤ 0.6	3.817 (9.868–14.778)		0.692 (0.115–4.160)*	0.349 (0.129–0.974)*	4.432 (0.427–1.163)	1.219 (.0.598–1.530)
0.7 < ADC ≤ 0.8	5.752 (2.178–15.198)		1.658 (0.376–7.304)*	0.798 (0.476–1.232)*	7.816 (0.920–1.335)	0.553 (0.181–0.910)*
0.8 < ADC ≤ 0.9	1.121 (4.730–2.658)*		5.999 (1.761–20.428)	0.898 (1.497–10.687)	1.632 (3.132–8.499)	0.990 (0.202–1.392)*
0.9 < ADC ≤ 1.0	0.651 (0.914–1.716)*		1.231 (0.339–4.466)	1.452 (3.523–5.986)*	0.608 (0.147–1.705)*	0.802 (0.779–1.252)*
1.0 < ADC ≤ 1.1	3.906 (0.879–1.860)		3.171 (0.339–4.466)	1.501 (2.009–3.452)*	3.917 (3.890–7.998)	1.054 (0.371–1.529)*
1.1 < ADC ≤ 1.2	2.925 (1.334–6.776)		2.496 (7.886–9.009)	1.453 (1.650–2.221)*	1.595 (3.435–4.667)	0.510 (3.445–8.956)*
1.2 < ADC ≤ 1.3	1.334 (6.004–9.657)*	Reference	0.399 (0.111–1.433)	1.562 (1.312–9.673)*	0.110 (0.005–1.127)*	0.849 (0.445–1.956)*
1.3 < ADC ≤ 1.4	1.679 (0.679–1.679)		1.957 (0.598–6.410)	1.024 (0.034–1.024)	4.008 (0.008–0.995)	1.389 (0.672–1.334)
1.4 < ADC ≤ 1.5	1.355 (0.066–1.445)		1.769 (0.998–1.884)	1.886 (0.554–0.981)	0.776 (0.549–1.886)*	0.679 (0.630–1.086)
Gene variation						
p32 deletion	0.182 (2.397–35.169)		0.678 (2.586–4.089)*	3.725 (2.245–4.682)	1.733 (1.198–4.990)	0.645 (0.009–1.879)*
P53 deletion	0.053 (1.609–10.206)*		0.942 (2.847–16.927)	3.725 (0.242–1.535)	0.359 (0.867–1.007)*	1.828 (1.905–3.445)*
RB1 deletion	1.600 (0.856–2.991)		2.104 (1.072–4.129)	1.600 (0.768–3.331)	0.290 (0.065–2.005)	1.492 (0.067–1.992)*
q21 amplification	0.050 (1.089–3.860)*		1.319 (0.639–2.723)**	4.239 (2.047–8.779)*	1.632 (0.494–5.392)	0.985 (0.181–5.355)*
IGH rearrangement	0.640 (0.278–1.475)		0.824 (0.332–2.045)	0.205 (0.074–0.565)	0.993 (0.072–2.067)*	0.346 (0.064–1.729)*

*ADC* apparent diffusion coefficient, *CR* complete response, *PD* progressive disease, *PR* partial response, *sCR* strict complete response, *SD* sable disease, *VGPR* vary good partial response

*p* value for the model is less than 0.001. A single asterisk (*) implies statistically significant

## Discussion

This is a retrospective study that explores the hypothesis of whether or not clinical variables and FISH-derived metrics are associated with the ADC value of WB-DWI in patients with MM. Our results indicate a correlation between the ADC value and genetic variation and suggest that both are related to the clinical outcome of MM patients. In contrast to previous studies, this study investigates not only the clinical indicators that affect the ADC value of WB-DWI but also the relationship between ADC value and MM treatment response. It is worth mentioning that it also includes the derivative results of FISH detection, which are not addressed in previous studies. However, due to its retrospective nature, the results of the current study need to be validated by future research. In our results, FISH-derived metrics and ADC may have significant roles in improving the comprehensiveness of prognosis as well as in guiding therapeutic options in MM.

Whole-body CT, PET/CT, and other examination methods are applied to MM bone diseases, and they have good detection capabilities in cortex, medulla and extramedullary diseases [17]. Compared with these methods, WB-DWI has its merits and limitations. WB-DWI, with its advantages of large imaging range and no radiation, especially the ADC value which can be used to quantify the diffusion of water molecules in tissues, has become an alternative method for the detection of bone disease in MM patients [5, 18]. Limitations of WB-DWI include possible difficulties in focus segmentation in patients with multiple bone marrow diseases [5]. WB-DWI is also not satisfactory for distal extremity and extramedullary lesions. However, WB-DWI has been shown to produce good consistency in the detection of bone marrow and extraosseous metastasis (κ = 0.887, agreement = 94.44%, *p* = 0.001) [19].

Although the role of ASCT in improving the overall survival in older patients (age ≥ 65 years) is still uncertain [20], older patients are included in the study to ensure an adequate age span. The ADC value of intramedullary lesions is affected by many factors [21]. The quantified ADC variations of the brain and pancreas provide standard reference points for radiologists [22]. It is inferred that age also plays an important role in the ADC value of MM. However, we find no correlation between the ADC value and patient’s age.

In MM patients, decreased serum albumin levels are considered to be associated with poor prognosis, although the cause is unclear [23]. The albumin level is mainly determined by the stage of MM disease or tumor load and has prognostic significance [23]. Serum LDH is widely distributed in the body and increases with tumor proliferation. In addition, experts believe that relapse of MM may be considered aggressive if there is the presentation of several features including elevated LDH [14]. It has been reported that high serum LDH is associated with advanced disease and low survival rate in elderly patients with MM. Studies have found that the 5-year survival rate of patients with LDH levels higher than the upper limit of normal (LDH > 260 IU/L) before transplantation is less than 5%, while the survival rate of patients with lower LDH levels is 22% [24]. These results suggest poor prognosis in patients with increased LDH and decreased albumin. As a result, it is reasonable to believe that albumin and LDH level are related to the activity of myeloma cells. Contrary to what would be expected from the results, albumin is only negatively related to the ADC value after chemotherapy as the ADC_4c_, and the levels of serum LDH and albumin are not related to the change of ADC value.

Serum β 2-MG is a strong prognostic factor and is considered to be related to tumor burden [13, 23]. The increase in β2-MG is an important predictor of overall survival; it cannot, however, predict progression-free survival, where the increase in β2-MG reflects the increase in total disease burden rather the reaction to chemotherapy that may show initial sensitivity and later recurrence of the disease [25]. In accordance with the above results, there may be a certain correlation between the β2-MG level and ADC_0_, but no significant correlation between β2-MG and ADC_4c_. This result may be due to the complicated sources of serum β2-MG after treatment. In addition to the abnormal proliferation of plasma cells, which are responsible for the secretion and synthesis of β2-MG, necrotic cells also release a large amount of β2-MG [26]. More than that, A retrospective study of thirty patients with MM suggest that the β2-MG level may be inconsistent with Whole-body MR imaging, especially in advanced MM [13]. The level of β2-MG in serum fails to reflect the real tumor load after treatment, on the basis that it can have different sources other than tumoral lysis, and this is the reason why no correlation is found between β2-MG levels and ADC_4c_.

ISS and RISS stages reflect the disease burden of MM [27, 28]. Soekojo et al. [29] reported that when compared with the RISS, there was a high level of concordance in high-risk patients identified using FISH analysis with an independent cohort of 375 patients. It can be deduced that the RISS staging as well as the FISH results are good indicators of patient prognosis. FISH plays an important role in the prognosis and risk stratification of MM patients [10]. FISH test results may be as closely related to the burden of MM disease as the RISS staging [30]. ADC values have been found to be correlated with tumor growth and differentiation. In a study of retinoblastoma involving 72 children, the ADC value was positively correlated with the degree of tumor differentiation (r = 0.87, *p* = 0.007), and negatively correlated with tumor size (r = 0.68, *p* = 0.015) [31]. A study of nasopharyngeal carcinoma reported similar findings: the ADC value was found to be negatively correlated with the volume of the nasopharyngeal carcinoma (r = − 0.799, *p* = 0.03) [32]. In addition, it has been found that the ADC value is also related to the metabolism of the tumor. The level of CH/Cr in cervical squamous cell carcinoma is negatively related to the ADC value (r = − 0.662, *p* = 0.001) [33].

FISH is used in standard clinical workflows for the detection of chromosomal abnormalities to identify high-risk patients. The positive results of FISH often indicate rapid proliferation and poor differentiation of MM tumor cells. In MM patients with such potential features, the tumor cells in the lesions are more closely arranged, which affects the ADC value. From the microscopic changes of tumor tissue, it could be inferred the internal relationship between ADC value and tumor burden of MM. RB1 gene deletion is a common chromosomal abnormality in MM patients, which is caused by complete or partial deletion of chromosome 13. It can be detected in 40% of MM patients and is an important prognostic factor for early detection [11]. Interleukin-6 plays a central role in the pathogenesis of MM. The RB1 gene is thought to downregulate the level of interleukin-6 (IL-6) [34]. This hypothesis can reasonably explain the treatment outcome of RB1 prediction of the treatment outcome of PD.

The correlation between FISH-derived measures and ADC values in MM has not been previously evaluated. Cui et al. [35] found that specific sets of miRNAs could be considered as biomarkers for distinguishing MM with 1q21 amplification. Patients with 1q21 amplification are more likely to have abnormal amplification of RB1 and p53 than those without this condition. Chatonnet et al. [36] found that specific genes on 1q21 were involved in the transcription factor network that controls cell proliferation, which led to excessive proliferation of cancer cells, thereby affecting the ADC value of bone marrow lesions. It is well known that a considerable number of MM patients carry translocations of targeted genes, especially in the switch region of the IGH gene. These translocations juxtapose the oncogenes to the neighboring powerful IGH enhancer (5'-IGH), which drives the abnormal expression of the translocated oncogenes [8]. Studies have shown that IGH rearrangement can affect related genes and trigger the autocrine mechanism of tumors [37], leading to disease progression. This may be the cause of the increased tumor load that leads to a decrease in the ADC value. It has been suggested that IGH-MMSETMB4-2/MB4-3 transcripts of fusion genes in t (4;14) MM indicate a poor prognosis [38]. Regarding the negative correlation between ADC_0_ and IGH rearrangement observed in this study, the decrease in the ADC value is often indicative of a heavier disease burden. Not all studies support the association between ADC and genetic abnormalities. Yuan C et al. found no significant correlation between the ADC value and genetic variation [39]. However, whether the IGH rearrangement is related to disease progression needs to be investigated in a future study with larger samples.

Whether ADC values at the baseline visit (ADC_0_) can predict the outcome of patients is an intriguing question. MR-based texture analysis may be helpful for the evaluation of MM patients' response to treatment [40]. MRI-based texture features are expected to be used as an auxiliary tool to evaluate the prognosis of MM patients. In addition, this study has shown that the average ADC of patients in the CR/VGPR group increases significantly after induction chemotherapy [41]. However, Park et al. [42] considered that MR imaging was useful in the evaluation of PD (sensitivity 94.7%, specificity 84.2%), but the evaluation of CR was not satisfactory (sensitivity 4.5%, specificity 98.1%), which also involved the information of the ADC map. When the baseline ADC value is 0.808 × 10^–3^ mm^2^/s, ADC has the specificity of 68.05% and sensitivity of 54.09% in predicting the increase in ADC after treatment [43]. Determination of ADC before initiating treatment may predict the outcome of treatment to some extent.

Also, consistent with this, several investigators have reported the prognostic value of the ADC value in MM patients. This, however, has not been incorporated in any widely used criteria, although it is able to identify patients with genetic variations and other clinical parameters. Because the patient numbers in the present study are relatively small, further investigations are needed to clarify this matter. The content of this study mainly involves the ADC value of WB-DWI. Inclusion of the texture analysis of DWI images may provide more detailed information for diagnosis and prediction of outcomes. The existing MR texture features are related to the degree of MM bone marrow infiltration and some biochemical indicators. It needs to be further explored for the application of texture features in the treatment effect of MM. While this is a limitation of this study, it also offers a possible direction for the next step [40]. Combining the above factors with knowledge of the molecular pathways involved, this important information is critical for accurate MM diagnosis and for predicting the progress of the disease, and opens the way for extensive research on this topic.

## Conclusion

This study has shown significant correlations between the ADC value and FISH results as well as other clinical parameters including the treatment response. ADC values increase significantly after four-course induction chemotherapy. The change of ADC value was correlated with RISS stage, baseline serumβ2-MG level and IGH mutation. The baseline ADC value and IGH rearrangement may be the independent risk factors for MM treatment outcome. The baseline ADC value less than 0.4 × 10^–3^ mm^2^/s, combined with p53 or RB1 deletion, may indicate that the treatment response tends to be unsatisfactory. However, additional research is needed to further define the inter-relationships among the ADC value, FISH results and the other clinical indicators.

## Data Availability

The datasets generated during and/or analyzed during the current study are available from the corresponding author on reasonable request.

## Abbreviations

β2-MG: β2-Microglobulin
ADC: Apparent diffusion coefficient
ADC_0_: ADC value at baseline visit
ADC_4 c_: ADC value at 4-course induction chemotherapy follow-up
ASCT: Autologous stem cell transplant
CR: Complete response
FISH: Fluorescence in situ hybridization
iFISH: Interphase fluorescence in situ hybridization
IMWG: International Myeloma Working Group
ISS: International staging system
ROI: Region of interest
LDH: Lactic dehydrogenase
MM: Multiple myeloma
PD: Progressive disease
PR: Partial response
RISS: Revised international staging system
sCR: Strict complete response
SD: Sable disease
VGPR: Vary good partial response
WB-DWI: Whole-body diffusion-weighted imaging
